# Effect of Shock-Induced Cavitation Bubble Collapse on the damage in the Simulated Perineuronal Net of the Brain

**DOI:** 10.1038/s41598-017-05790-3

**Published:** 2017-07-13

**Authors:** Yuan-Ting Wu, Ashfaq Adnan

**Affiliations:** Mechanical and Aerospace Engineering, the University of Texas at Arlington, Arlington, 76010 USA

## Abstract

The purpose of this study is to conduct modeling and simulation to understand the effect of shock-induced mechanical loading, in the form of cavitation bubble collapse, on damage to the brain’s perineuronal nets (PNNs). It is known that high-energy implosion due to cavitation collapse is responsible for corrosion or surface damage in many mechanical devices. In this case, cavitation refers to the bubble created by pressure drop. The presence of a similar damage mechanism in biophysical systems has long being suspected but not well-explored. In this paper, we use reactive molecular dynamics (MD) to simulate the scenario of a shock wave induced cavitation collapse within the perineuronal net (PNN), which is the near-neuron domain of a brain’s extracellular matrix (ECM). Our model is focused on the damage in hyaluronan (HA), which is the main structural component of PNN. We have investigated the roles of cavitation bubble location, shockwave intensity and the size of a cavitation bubble on the structural evolution of PNN. Simulation results show that the localized supersonic water hammer created by an asymmetrical bubble collapse may break the hyaluronan. As such, the current study advances current knowledge and understanding of the connection between PNN damage and neurodegenerative disorders.

## Introduction

Cavitation is the term defined by bubbles that are created when pressure is dropped below saturated vapor pressure. In principle, cavitation-induced bubbles are unstable in nature, that is, as soon as the pressure is reinstated above vapor pressure, bubbles immediately collapse. The high-energy jet produced during cavitation collapse often causes surface damage to many high-speed machines, such as underwater propeller blades. Recently, a series of studies have been conducted focusing on a similar damage mechanism of collapsing nanobubbles in the biophysical systems^1, 2^, especially in the most delicate system—the human brain^3–5^.

**Figure 1:**
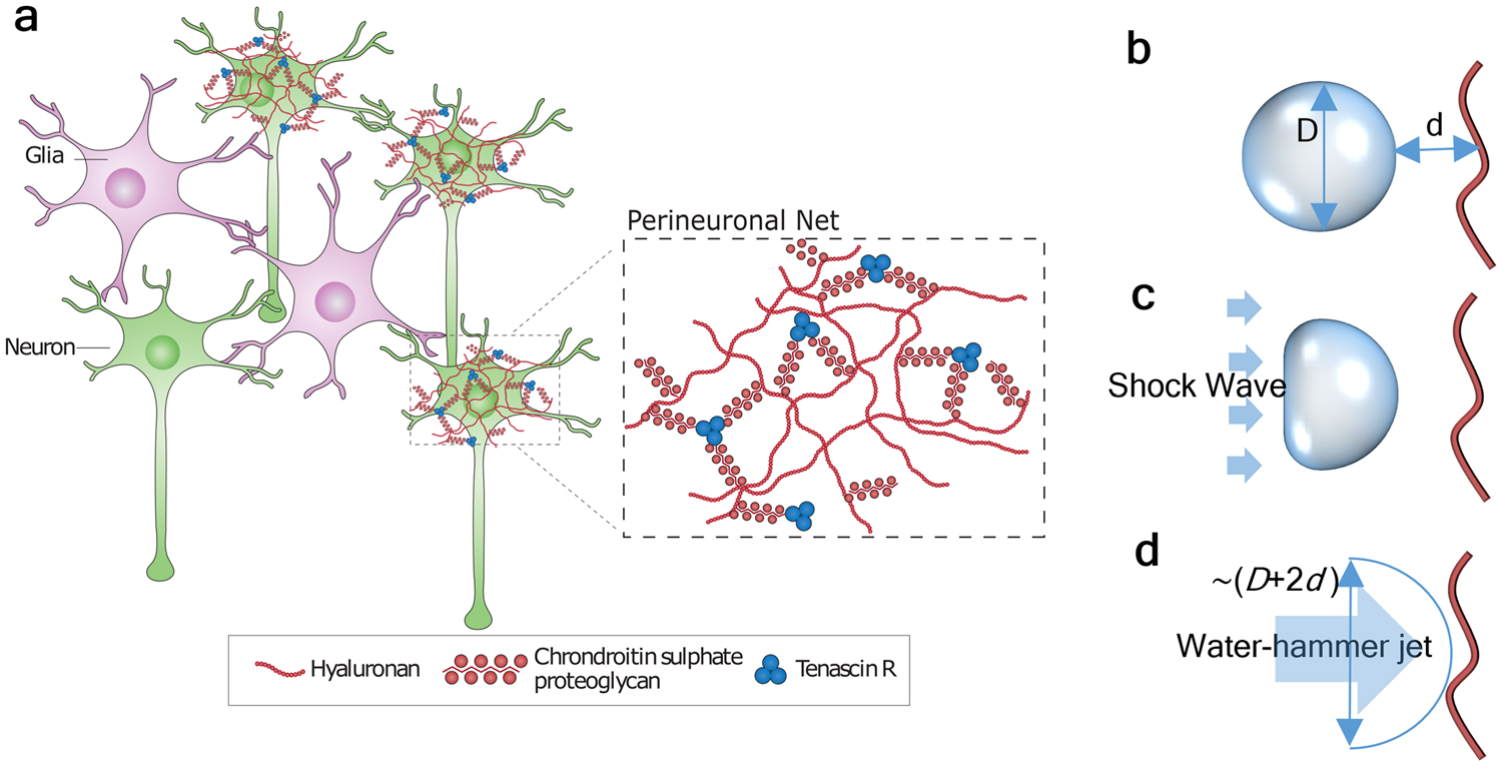
(**a**) Neurons surrounded by the ECM in the CNS. The region in ECM ﻿in the immediate vicinities of neurons are called Perinuronal Net (PNN). ﻿The components of PNN are shown in the magnified view (adapted from Fig. 1 of ^37^﻿) (Permitted reprint) and (**b**–**d**) Schematic of pre-, during, and post-collapse bubble.

A blast from an explosive device may lead to traumatic brain injury (TBI)^6^, and the level of injury can range from a mild concussion to a severe penetrating injury. Recent studies on animal models suggest that mild TBI^7–9^ is directly connected with the later appearance of progressive neurodegenerative disorders such as Alzheimer’s disease (AD)^10–13^ and chronic traumatic encephalopathy (CTE)^14^ as well as posttraumatic stress disorder (PTSD)^15–18^. Under a blast, the brain suffers from a highly dynamic mechanical force, and, in principle, pressure in some portion of the brain’s fluid system may become low enough to induce cavitation. Until now, most experimental studies mimicking blast-like scenarios are observed via optical microscopy, which are limited by the device resolution being 1 μm or higher^2, 8, 19–21^. Of these studies, only a handful of studies have been reported on how different micro-scale brain components, such as cell membranes (lipid bilayers)^2, 22–29^, ion channel^30^, and blood-brain barrier^31, 32^, respond to impact caused by cavitation bubble collapse. While these studies explored various components of the brain when subjected to a shock-wave and cavitation collapse, little is known about the role of a shock-wave on the morphological evolution of a brain’s extracellular matrix (ECM)^19^. Animal studies suggest that many neurodegenerative disorders are associated with the pathological changes of the ECM that primarily surrounds the neuron cells (Fig. 1a)^33–37^. The area of ECM surrounding the neurons is known as a perineuronal net (PNN)^11, 12, 38–42^. Structurally, the PNN is primarily composed of water, free ions and three major types of biomolecules: hyaluronan (HA), tenascins, and lecticans (Fig. 1b)^37, 43^. From a physiological standpoint, pathological changes of PNN would imply degeneration or transformation of its biomolecular constituents.

While it is not clearly known what level of applied shock on a PNN will lead to the formation and collapse of a cavitional bubble, it is known that the nanobubbles are generally unstable, and surface tension at the nanometer scale is a critical factor. Based on the Young-Laplace law^44^, the Laplace pressure from surface tension inversely scales with the bubble size^45, 46^. At the nanometer scale, when a bubble containing fluid is impacted by a traveling wave, the local fluctuation of pressure in the fluid combined with surface tension significantly increases the collapsing possibility of nanobubbles. During collapse, liquid surrounding a bubble is driven toward the center of the bubble resulting in the conversion of potential energy to kinetic energy. If the bubble collapse is asymmetrical^47^, then it may induce a high-velocity jet, referred as “water hammer (WH)”. A WH is characterized by its one-direction flow pattern and has the potential to cause damage to materials coming its way. Asymmetrical collapse can occur based on two possible scenarios. The first scenario is associated with the “Rayleigh collapse” where the cavitation-bubble collapses under a uniformly reinstated pressure near a solid surface to guide the water hammer toward the solid surface. The second scenario is connected to a shockwave induced bubble collapse where the water hammer is formed and travels along the shockwave propagating direction^48^. In both scenarios, the pressure difference drives the water hammer. In the Rayleigh collapse, the pressure difference exists between the inside and outside of the bubble. For shock-induced collapse, the pressure difference is created due to variation between the post-shock pressure and the bubble pressure. It can be argued that, in principle, blast-induced shock can cause formation and collapse of cavitation bubbles in PNN, and the resulting jet produced near the collapsing nanobubbles can become high enough to cause pathological changes of PNN.

In this paper, fully atomistic simulations were used to interrelate applied shock, water-hammer kinetics and damage mechanisms in PNN. We investigated whether water hammer is even capable of breaking the HA. A study on the effect of cavitation bubble size on HA damage is also presented. To investigate these issues, we used reactive molecular dynamics simulation (reaxFF potential)^49, 50^ to model the events occurring at the nanometer scale and sub-nanosecond time frame. While conventional molecular dynamics simulation can capture events like bubble ripening^51–53^ and collapse^23, 24, 30, 31, 54–58^ in homogenous fluids, the use of reactive molecular dynamics is essential to the assessment of PNN damage because of PNN’s heterogeneous morphology. The simulation results demonstrate the possible shock-induced damage mechanisms of HA due to bubble collapse.

## Simulation Methods

Our model consists of three major components: Hyaluronan (HA), surrounding water, and ions. The initial molecular structure of HA is obtained from prior molecular dynamics simulation suggesting that a stable periodic formation of HA is a four-fold helix^59^. Since the available profile of HA only has the acidic part, i.e. the biomolecule in water is not electronically neutral, for each simulation, we had to balance the “extra” charges with counter ions. Here we used a matching number of H ions (H+) as counter-ions located 4 nm away from the symmetric axis (5 for each fold, 1 for the end node). The size of the simulation box is approximately 16 nm by 16 nm by 25 nm containing 599,205 atoms in total with a 12-fold hyaluronan (~500 atoms). Once the model was developed, we equilibrated the whole system at 300 K (26.85 °C) and 101 kPa pressure (Appendix 1 has more detail on the simulation set-up). We used Nose-Hoover’s barostat (piston) and thermostat (heater). The time constants for the piston was set at 1 ps and the heater was set at 50 fs.

Our simulations were aimed to quantify the impact of bubble collapse triggered by a shock wave (Fig. 1d). To create a “cavitation-induced bubble,” we manually removed a water sphere from the original simulation box. The artificially created vacuum is slightly different from the naturally formed cavitation-induced bubble in that a naturally formed bubble contains gas molecules inside. In our prior simulations (Appendix 2), we found that a cavitation bubble encloses only low-pressure gas. Therefore, we determined that the number of gas molecules inside the bubble are too few to affect the shock collapse process. We considered three different bubble diameters, 5 nm, 8 nm, and 10 nm. One model was also built without a bubble. We generated shock from the positive z-direction toward the negative z-direction using the reflective boundary condition method^56, 60^ (Appendix 1 describes the shock generation method). Shock propagation and bubble collapse were simulated with an NVE ensemble. The z-direction was selected as the shock propagating direction. Reflective boundary condition is applied in boundaries normal to z direction. The other two boundaries (normal to x and y direction) are modeled as periodic. The shock front travels such that it first encounters the cavitation bubble. As the bubble collapses, the traveling shock front then hits the HA with the collapse-induced WH. In this paper, we have considered initial “piston” velocity (post shock particle velocity) *v*_*p*_ as 1 km/s, 2 km/s and 3 km/s, which translates to shock velocity *v*_*s*_ 3.6 km/s, 5.35 km/s and 7.2 km/s, respectively.

By subjecting the four distinct models (i.e. models with three different bubble sizes plus the one without a bubble in PNN) with three different shock wave velocities, we performed simulations under 12 different scenarios. Three independent sets of simulations for each scenario (total 36 sets) were conducted to eliminate any significant statistical disparity. The initial orientation of the HA molecule along with its axis is the only factor that varied in the three sets of simulations. The HA was rotated 90 degrees around its length axis for each set before the equilibration. We recognize that the size of the bubbles is limited based on the nm length scale. Prior research^61–63^ shows surface or bulk bubble of a few tens of nanometers were stable for a few hours. Unfortunately, the computational cost for high fidelity MD simulation using reactive potential on systems with the “larger” bubble is very high and beyond the scope of current effort.

Maximum over-pressure generated from an explosive blast is ~1 MPa. Since we are simulating bubbles that are about 10 times smaller than the experimentally observed stable nanobubbles, we chose the shock velocities related to a similar amount of WH kinetic energy. The total water-hammer-jet kinetic energy is related to the bubble size and the post-shock pressure of an ideal planar shock wave through:

1 $${E}_{k}\propto {D}^{3}{p}_{p}$$

where *E*_*k*_, *D*, and *p*_*p*_ are the total kinetic energy of the water hammer, diameter of the bubble, and post-shock pressure, respectively. It is apparent that we need to have *p*_*p*_ to be 1000 times stronger than 1 MPa (i.e. ~1 GPa) to remain consistent with the microscopic bubble (10 times larger than what we used) collapse.

The interatomic and intermolecular interactions for the entire system, including HA, water, and counter ions, have been prescribed by high-fidelity but computationally expensive ReaxFF potential^49^. Unlike conventional interatomic potential where chemically bonded interatomic interactions are estimated based on a predefined potential function, the ReaxFF potential determines interatomic bonding as the outcome of atom position at each time step, which makes it capable of simulating bond breaking and building. The use of ReaxFF in our simulation was essential for assessing the damage to molecules such as HA that actively interact with surrounding water molecules and ions. All of our simulation were conducted on LAMMPS^64^. Post-processing and visualization of data were conducted using MATLAB^65^ and Ovito^66^, respectively.

## Results and Discussion

**Figure 2:**
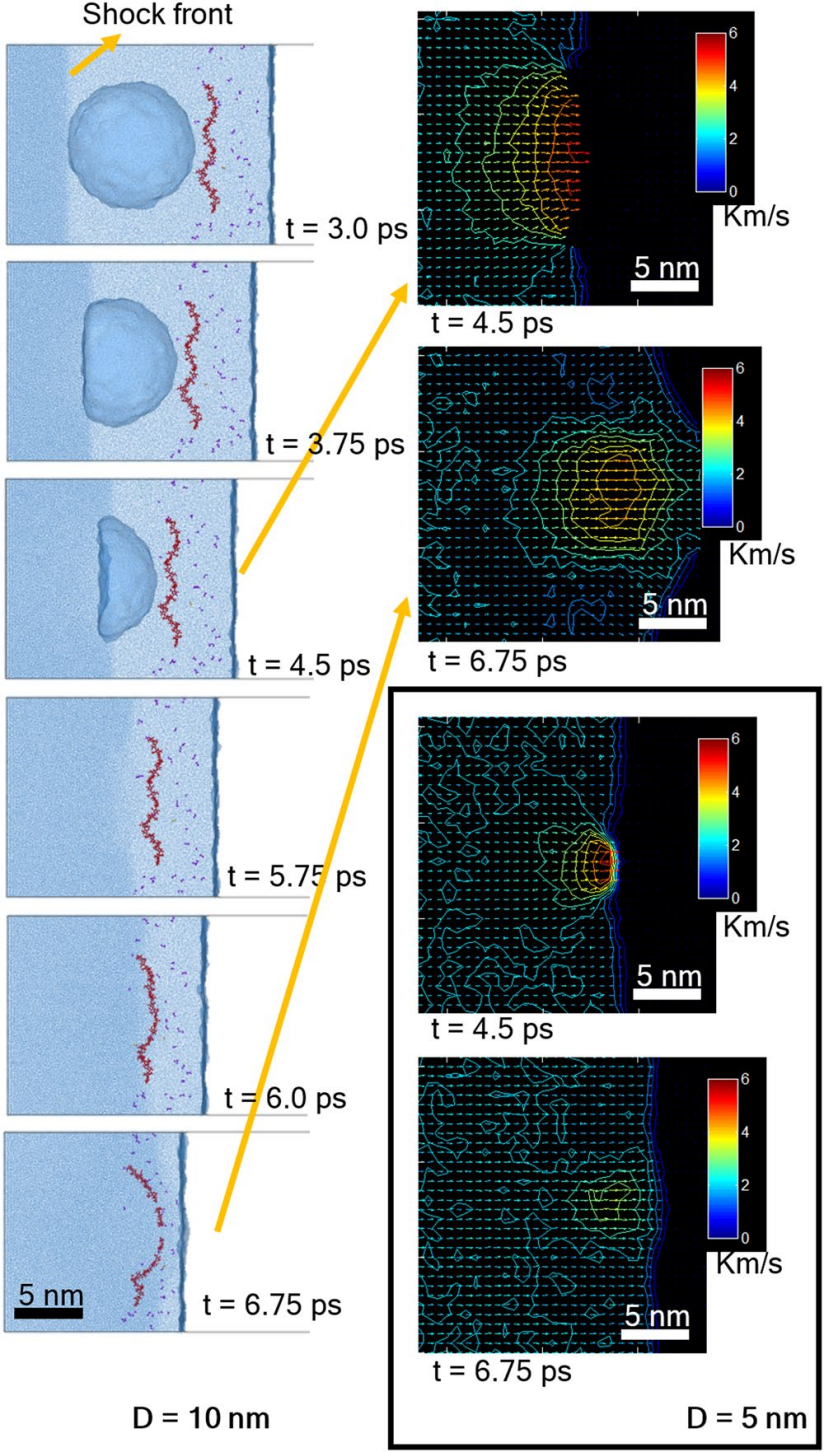
Cavitation-collapse triggered by shock, shock velocity = 5.35 km/s, bubble radii = 10 nm (top two) and 5 nm (bottom two). Scale for velocity color map is Km/s.

The bubble collapse process and the subsequent effect on the HA is shown in Fig. 2. The mechanism of bubble collapse agrees well with many existing simulation studies^23–25, 30, 31, 55, 56, 60^. It can be inferred from Fig. 2 that when a shockwave passes through the cavitation bubble, the post-shock pressure gradually compresses the anterior side of the bubble. Since the density of a bubble’s interior is far less than the surrounding bulk liquid, the post-shock pressure leads to faster bubble collapsing speed with a magnitude even faster than the shockwave. Subsequently, the collapsing jet (WH jet) strongly collides with the other side of the bubble. It can be inferred that larger bubble collisions create stronger WH jets, which hold larger kinetic energy. Such a WH jet takes longer to dissipate after the collision. Once the WH jet lands on the HA, the HA starts to deform and continues to deform until the WH jet completely passes through it. The GIF animation of bubble collapse, velocity contour, pressure contour, and density contour can be found online as Supporting Material.

**Figure 3:**
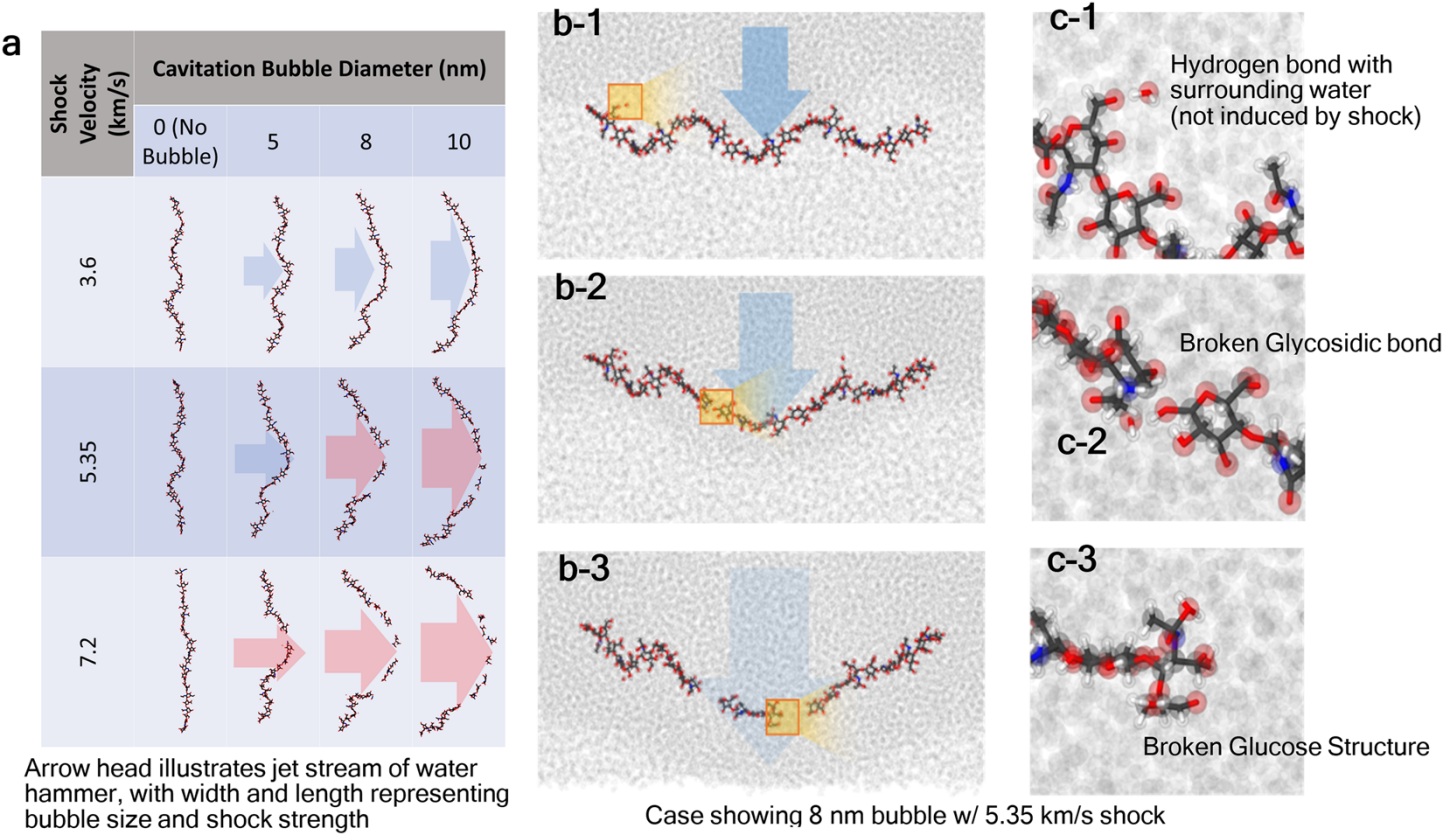
Atomistic simulation snapshots showing (**a**) evolution of HA impacted by WH jet. For the cases of broken HA, the WH jet streams are marked in red; otherwise, they are marked in blue (**b**) a magnified view of the ruptured HAs, and (**c**) damaged location of the ruptured HA.

Depending on bubble size and initial shock-speed, HA’s structural evolutions inside PNN can be described either by (a) large-scale deformation or (b) complete rupture, as shown in Fig. 3(a). In Fig. 3, the “arrow” used to indicate the WH jet direction is marked in blue if the HA is intact. For an HA broken by a cavitation collapse, the “arrow” is marked in red. The five cases where HA was broken were due to the presence of larger bubbles and faster shockwave velocities. For a 10-nm bubble case, the rupture can even happen in multiple sites. It can be hypothesized that HA has no time to release the stress when moving along the axial direction. In other cases, the HA is seen to sustain bending-like large deformation but remains intact. In those cases, the HA can stretch its helical structure to avoid breaking.

The local bonding evolution in the form of chemical, hydrogen and the van der Waals bonding of HA is revealed in Fig. 3(b,c). Three major events can be observed. First, due to the acidic nature, HA keeps building and losing hydrogen bonds with the surrounding water molecules. There is no significant increase or decrease of hydrogen bonding before and after shock and jet stream. Second, a strong jet stream can rupture the HA into two or more pieces. As shown in Fig. 3c, the first breaking point of HA involves breaking of a glucoside bond. In this case, the “intact” segments of HA do not exhibit major structural change. In the last scenario, the glucose ring structure in HA can also be broken during jet impact.

**Figure 4:**
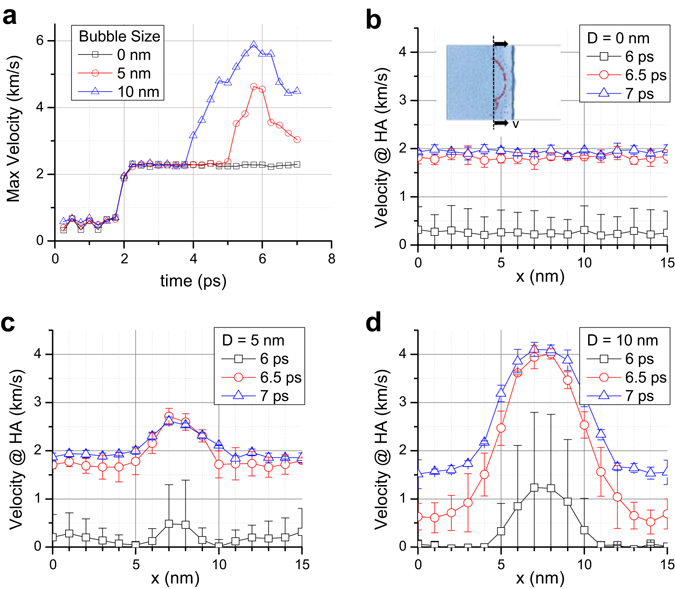
For shock velocity of 3.6 km/s, (**a**) graphs the maximum local velocity profile with time, and (**b**–**d**) graphs the local velocity along the HA axis. The local velocity has been computed by taking average velocities of atoms inside a cubic bin where side equals 1 nm. Error bar shows the relative local velocity variation based on the three simulations conducted with the same bubble size and the same applied shock velocity.

**Figure 5:**
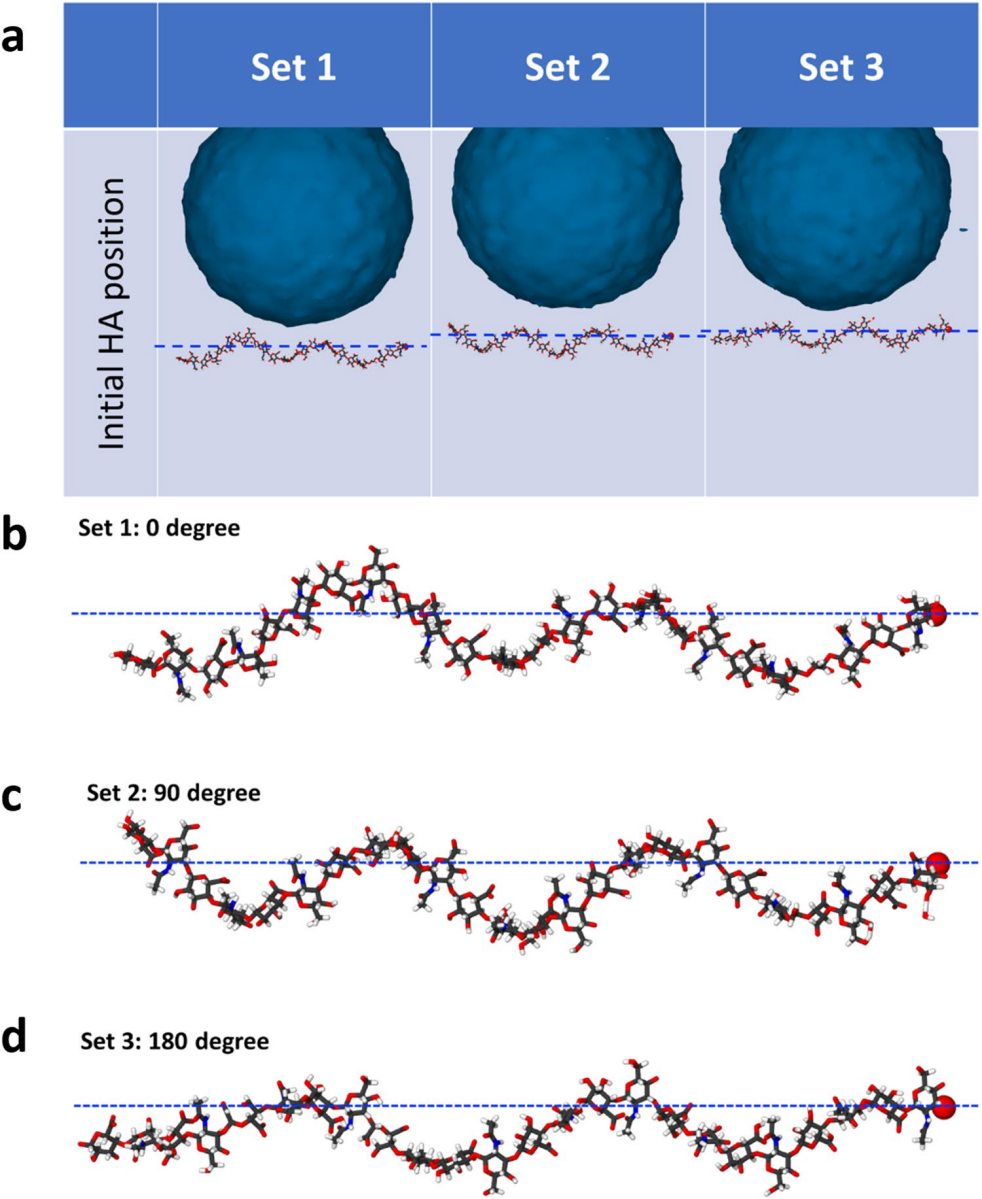
(**a**) Equilibrated MD snapshots showing the variation in the distance between HA and bubble surface caused by the change in HA’s axial orientation. Note that the axial orientation of HA varied by 90 degrees between the three sets of simulation. (**b**–**d**) Magnified MD snapshots of the HA in set 1, 2 and 3. The atoms are colored as: white for H, black for C, blue for N, red for O.

To discuss the damage mechanism of HA further, we picked up the systems with 5 nm and 10 nm bubbles that are subjected to the same shock velocity of 5.35 km/s. We divided the simulation cell into small cubic bins (each bin has a side length = 1 nm) and evaluated the continuum properties inside. We chose the 5 nm and 10 nm bubble diameters because same shock velocity on 5 nm and 10 nm bubbles yielded two different outcomes—one breaks the HA and the other does not. It is interesting to observe that in the case of a 5 nm bubble, the max velocity drops much quicker compared to the case of a 10 nm bubble. The local velocity profile can be found in Fig. 4. As shown in Fig. 4(a), as soon as the bubble starts to collapse, the maximum local velocity raises, and then it starts to drop once the jet hits the other side of the bubble. Figure 4(d) shows the case of a 10 nm bubble where WH ruptures HA. It can be observed that WH velocities near the center and the ending of HA differ by ~2.5 km/s. No velocity difference is observed when no bubble is present (Fig. 4(b)). The velocity difference is less than 1 km/s when bubble size is 5 nm, as can be seen in Fig. 4(c). At any particular instant of time (e.g. 6 ps, 6.5 ps or 7 ps) and at any particular point “x” (0 nm ≤ × ≤ 15 nm), local fluid velocities vary among the three simulation sets. Error bars at each data point indicate the standard deviation. We believe two factors have played role in local velocity variation. One is due to the local dynamics of individual atoms. Since local fluid velocities at any location are calculated from the sum of atomic velocities, the stochastic nature of atomic velocities is reflected in the local velocity estimation. The relative variations in local velocities are also believed to be influenced by the axial orientation of HA relative to the oncoming shock front. Since we rotated HA about its own axis by 90 degrees when we build model for simulation set 2, and then by another 90 degrees (i.e. 180 degree rotation compared to the orientation in set 1) in simulation set 3, the shortest distances between the HA and the bubble surface slightly change, as shown in Fig. 5. Such changes in distance implies shock front lands on HA at slightly different time. However, we report local velocities at a particular instant of time only. As such, local velocities in different simulation set very at the beginning (i.e. at 6 ps). It can be noticed that the degree of deviation reduces once the HA is fully in the thrust zone. It can also be observed that the overall trends in local velocity profiles are very consistent confirming our simulation outcome is reproducible.

It is shown in Eqn. (1), the total water-hammer-jet kinetic energy is roughly proportional to the bubble size and the post-shock pressure. Since the Kinect energy disperses in the radial direction after the impact (see Fig. 1(d)), the averaged kinetic energy per frontal area that acts on the HA can be estimated by

2 $$\frac{{E}_{k}}{{(D+2d)}^{2}}\propto \frac{{D}^{3}}{{(D+2d)}^{2}}{p}_{p}$$

where *d* is the distance between the bubble and the HA. We have listed the “estimated” kinetic energy per frontal area for all nine cases (having a bubble) of our simulation in Table 1 using *d* = 1 nm. We regard this kinetic energy as “estimated” because the post-shock pressure was “estimated” based on spaced-averaged (post-shock area) simulation data obtained during the equilibration phase of our simulation. The magnitude of the pressure value will vary if someone space-averages the pressure value at different instant of time. As such, the data in Table 1 should be taken as a guideline for obtaining first-order estimated kinetic energy threshold to break the HA. Nevertheless, it can be inferred from Table 1 that the HA can break when energy exceeds 40 N/m.

**Table 1:** Theoretical estimation of water-hammer kinetic energy per area, The color scheme and the table arrangement are the same as shown in Fig. 3.

Shock velocity (km/s)	EstimatedPost-shock pressure, *p*_*p*_ (GPa)	Theoretical water-hammer kinetic energy per area, $\frac{{{\boldsymbol{D}}}^{{\bf{3}}}}{{({\boldsymbol{D}}{\boldsymbol{+}}2{\boldsymbol{d}})}^{{\bf{2}}}}$ (N/m)		
		D = 5 nm	8 nm	10 nm
3.60	2.65	6.76	13.5	18.4
5.35	7.97	20.3	40.8	55.3
7.20	16.2	41.3	82.9	112

For all simulations, the evolution of the molecular composition of HA and ions were monitored. We observe that bubble collapse does not induce significant composition change to the HA (except the rupture). Also, the ion count was not affected by the collision.

## Conclusions

By observing how the HA suffered from the water-hammer, it is obvious that the local damaging of the PNN was largely enhanced by the jet formed during bubble collapse. This is further corroborated from the case with no bubble where we found that even a high-velocity shockwave was not enough to break the HA. Thus, we conclude the following

- The results of the research presented herein suggest that larger the bubble size, the bigger the impact is on the HA. Unfortunately, the computational cost for the reactive MD is excessively high (a large memory is required). As such, bubbles larger than 10 nm were not tested in this study. The critical size of a bubble that is able to break HA with minimal pressure drop (post-shock and bubble pressure in this case) is still unknown.
- The estimation method of kinetic energy per frontal area gives a rough guideline for estimating the energy that is required to break HA. The estimation method developed in this research worked for the nine cases tested.

The link between blast induced Traumatic Brain Injury (TBI) with the PNN through the cavitation collapse is a very interesting research direction that can possibly relate to the symptoms of PTSD. If the HA can be broken by a nanobubble collapse, it is highly possible that a blast can disassemble the PNN. More studies on this possibility are underway.

## Electronic supplementary material

supplementary information
