# Supplementary material for: Effect of Shock-Induced Cavitation Bubble Collapse on the damage in the Simulated Perineuronal Net of the Brain
Source: Sci Rep. 2017 Jul 13;7:5323. doi: 10.1038/s41598-017-05790-3 (PMC5509702; doi:10.1038/s41598-017-05790-3)
Supplement: Supplementary file 1 — supplementary information [file 41598_2017_5790_MOESM1_ESM.pdf]

Effect of Shock-Induced Cavitation Bubble Collapse on the damage in the Simulated Perineuronal Net of the Brain.

Yuan-Ting Wu and Ashfaq Adnan*

Mechanical and Aerospace Engineering, the University of Texas at Arlington, Arlington, 76010, USA
*aadnan@uta.edu

Appendix 1: Simulation Set-up

The detail of our simulation set-up is described in this section.

1. Equilibration: The initial size of the simulation box is 16 nm, 16 nm, and 25 nm containing a total of 599,205 atoms. We have equilibrated the whole system for 60 ps. In the first 10 ps of equilibration, we kept the hyaluronan “fixed” but released this constraint for the next 50 ps of simulation. The equilibration was performed with NPT ensemble (fixed number of atoms, fixed pressure, and fixed temperature) at 300 K and 101 kPa. The temperature and pressure are controlled by using “fix npt” command in LAMMPS[1]. This command performs time integration on Nose-Hoover based equations of motion to generate positions and velocities sampled from the isothermal-isobaric ensemble. More details about the scheme and implementation can be found in LAMMPS website[1] and the linked references therein[2-6]. Specifically, the temperature is controlled by the thermostat that is applied to the translational degrees of freedom for the atoms. The external pressure is controlled by the barostat as scaler hydrostatic pressure. For prescribed values for thermostat and barostat time constants (we used 1 ps for the barostat and 50 fs for the thermostat), the time-averaged temperature and pressure of the atoms attain the target values (i.e 300 K and 101 kPa, respectively).

2. Shock Generation: We have generated shock using “reflective-boundary” method. First, we have replaced the periodic boundaries normal to z-axis to reflecting boundaries. We have generated vacuum between bulk atoms and +z direction (3 nm)/–z direction (7 nm) . We have then subjected the entire box of atoms to a velocity, v_p, along positive z-direction. The applied velocity v_p causes the entire box of atoms propel towards and eventually impact the reflective boundary at the same velocity v_p. Such impact on the reflecting boundary induces a shock wave on the impacting atoms. It is evident

from prior literatures that the physics of shock wave formed by reflection is same as conventional piston-driven shock wave formation. The shock velocity generated by our system agrees with experimental results and other simulation work.

Appendix 2: Cavitation Formation and Nucleation Site

In this section, we have investigated the distance between cavitation-induced bubble and hyaluronan to determine the minimum distance at which hyaluronan exhibit stable configuration. The simulation box containing water, hyaluronan, and ions are slowly stretched up to 23% in volume, which enables nano cavitation to form.

This set of simulation is aimed to obtain the characteristic distance between nanobubble and hyaluronan (Figure A1). The initial size of the simulation box is 16 nm, 16 nm, and 25 nm containing a total of 599,205 atoms with a 12 fold hyaluronan (~500 atoms), which is the same equilibrated box in the main article. We stretched the entire simulated domain, including all of the atoms, for 10.78% in volume (3.47% in length) within 10 ps. Then we continuued the simulation for another 40 ps with fixed doamin size. During the entire simulation, the temperature is fixed at 300K with the damping constant of 12.5 fs.

Figure A1 shows the results. It can be observed that when the box started to stretch, pressure starts to go down below atmospheric (i.e., building up of negative pressure or tensile pressure). Once the negative pressure is reached the critical pressure for bubble nucleation (in our case it is ~ - 170 MPa, which is close to the experimental result from[7]), bubbles start to form, and the pressure stops to build further even when the box is continued to expand. After the box stop stretching (10 ps), the pressure start to rise up. It can also be observed that the near-spherical bubbles are formed from many nucleation sites. Most bubbles, with an average size of ~2 nm, are seen to nucleate about ~3-5 nm away from the hyaluronan-liquid interface. The nearest bubble nucleation site we captured has the distance ~0.5 nm from bubble surface to the hyaluronan. At the end of the simulation (50 ps), all the cavitation-bubble tended to merge into one, which is the lowest energy state at given constraint.

By observing the interaction between the newly nucleated bubble and hyaluronan, it is clear that hydrophilic hyaluronan tends to repel the bubble away. The bubbles are only stable at a minimum distance of 0.5 nm from the hyaluronan implying

the presence of at least one or two layers of water between the bubble surface and the hyaluronan. The minimum distance of 0.5 nm is later used in the main article.

Appendix 3: Visual comparison of the three sets of simulation (with rotated HA as initial condition)

In Fig. A2, results of all three sets of simulations have been shown. The threshold of HA breaking are the same in all three sets. Since the HA of the three sets are rotated by 90 degrees each, the broken segment of the HA are at different location but following the same pattern of breaking glyosidic bond and breaking glucose structure (mostly for 7.2 km/s cases).

References

1 http://lammps.sandia.gov/doc/fix_nh.html (last accessed on 5-15-2017)

2 Plimpton, S. Fast parallel algorithms for short-range molecular dynamics. *Journal of computational physics* **117**, 1-19, doi:10.1006/jcph.1995.1039 (1995).

3 Shinoda, W., Shiga, M. & Mikami, M. Rapid estimation of elastic constants by molecular dynamics simulation under constant stress. *Physical Review B* **69**, 134103, doi:10.1103/PhysRevB.69.134103 (2004).

4 Martyna, G. J., Tobias, D. J. & Klein, M. L. Constant pressure molecular dynamics algorithms. *The Journal of Chemical Physics* **101**, 4177-4189, doi:10.1063/1.467468 (1994).

5 Parrinello, M. & Rahman, A. Polymorphic transitions in single crystals: A new molecular dynamics method. *Journal of applied physics* **52**, 7182-7190, doi:10.1063/1.328693 (1981).

6 Tuckerman, M. E., Alejandre, J., López-Rendón, R., Jochim, A. L. & Martyna, G. J. A Liouville-operator derived measure-preserving integrator for molecular dynamics simulations in the isothermal–isobaric ensemble. *Journal of Physics A: Mathematical and General* **39**, 5629, doi:10.1088/0305-4470/39/19/S18 (2006).

7 Azouzi, M. E. M., Ramboz, C., Lenain, J.-F. & Caupin, F. A coherent picture of water at extreme negative pressure. *Nature Physics* **9**, 38-41, doi:10.1038/nphys2475 (2013).

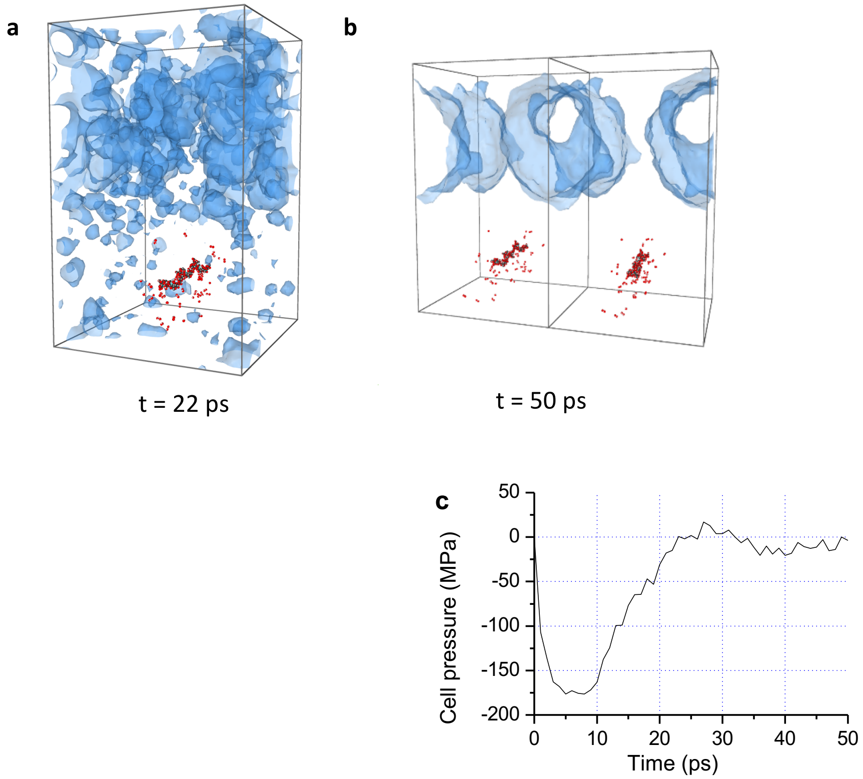

Figure A1. (a, b) Cavitation-induced bubble formation and coalescence. All water atoms except those on the bubble surface have been removed for clarity. The blue shades represent bubble surface. (c) Accumulated pressure in the simulation cell.

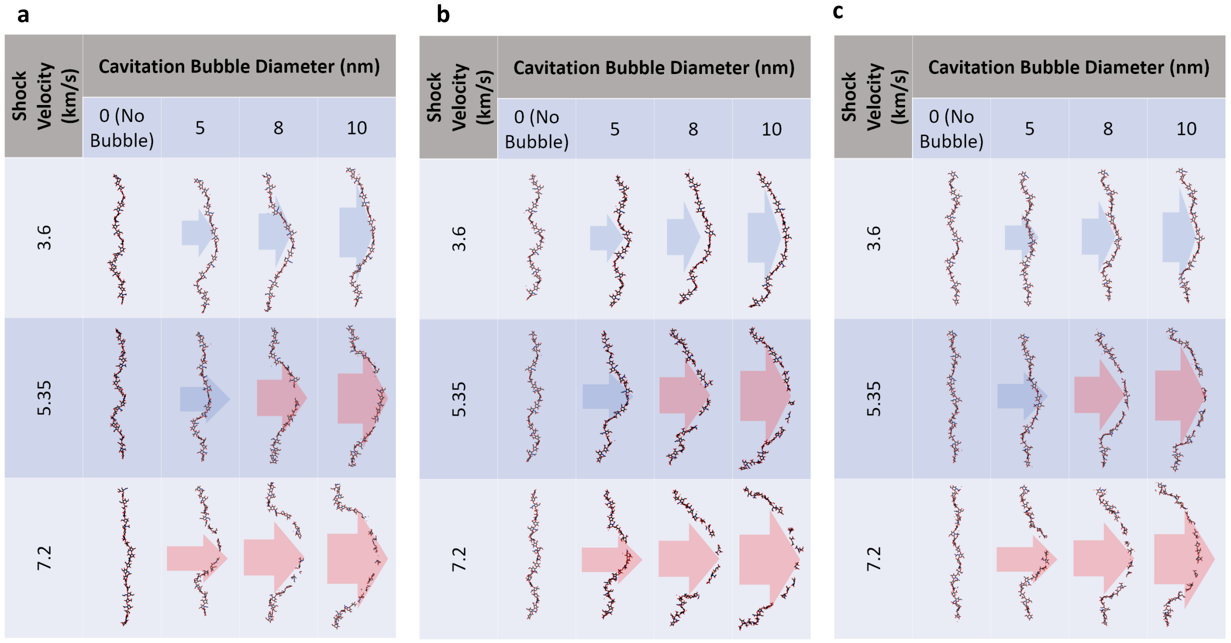

Figure A2. Atomistic simulation snapshots showing evolution of HA impacted by WH jet, based on results from simulation set (a) one (b) two and (c) three.
